# Supplementary material for: Analysis of Cholera Risk in India: Insights from 2017–18 Serosurvey Data Integrated with Epidemiologic data and Societal Determinants from 2015–2019
Source: PLoS Negl Trop Dis. 2024 Sep 3;18(9):e0012450. doi: 10.1371/journal.pntd.0012450 (PMC11398695; doi:10.1371/journal.pntd.0012450)
Supplement: S1 Table — (DOCX) [file pntd.0012450.s001.docx]

**S1 Table: Cholera incidence using vibriocidal cut off titre of 320 in selected 60 districts of India during past one year of blood collection**

| **Sate** | **District** | **Total Tested** | **Number of sera with reciprocal vibriocidal titer ≥ 320** | **Number of sera with reciprocal vibriocidal titer < 320** | **Elevated vibriocidal titre (≥ 320)** |
| --- | --- | --- | --- | --- | --- |
| Tamil Nadu | Vellore | 137 | 40 | 97 | 29.2 |
| Karnataka | Belgaum | 130 | 25 | 105 | 19.2 |
| Tamil Nadu | Coimbatore | 135 | 22 | 113 | 16.3 |
| Maharashtra | Akola | 93 | 15 | 78 | 16.1 |
| Uttar Pradesh | Allahabad | 131 | 21 | 110 | 16 |
| Madhya Pradesh | Tikamgarh | 116 | 18 | 98 | 15.5 |
| Madhya Pradesh | Sehore | 125 | 19 | 106 | 15.2 |
| Uttar Pradesh | Mau | 140 | 21 | 119 | 15 |
| West Bengal | Barddhaman | 124 | 18 | 106 | 14.5 |
| Rajasthan | Banswara | 125 | 18 | 107 | 14.4 |
| West Bengal | Dakshin Dinajpur | 127 | 18 | 109 | 14.2 |
| Madhya Pradesh | Shajapur | 144 | 19 | 125 | 13.2 |
| Andhra Pradesh | Krishna | 159 | 21 | 138 | 13.2 |
| Tamil Nadu | Karur | 148 | 19 | 129 | 12.8 |
| NCT of Delhi | North West | 134 | 17 | 117 | 12.7 |
| Rajasthan | Jaipur | 126 | 16 | 110 | 12.7 |
| Punjab | Bathinda | 143 | 18 | 125 | 12.6 |
| Maharashtra | Pune | 105 | 13 | 92 | 12.4 |
| Andhra Pradesh | Anantapur | 178 | 22 | 156 | 12.4 |
| Rajasthan | Barmer | 133 | 16 | 117 | 12 |
| Maharashtra | Osmanabad | 120 | 14 | 106 | 11.7 |
| Bihar | Patna | 105 | 12 | 93 | 11.4 |
| Bihar | Katihar | 144 | 16 | 128 | 11.1 |
| Odissa | Sambalpur | 162 | 18 | 144 | 11.1 |
| Rajasthan | Churu | 108 | 12 | 96 | 11.1 |
| Andhra Pradesh | Sri Potti Sriramulu Nellore | 144 | 16 | 128 | 11.1 |
| Karnataka | Uttara Kannada | 144 | 16 | 128 | 11.1 |
| Tripura | Dhalai | 119 | 13 | 106 | 10.9 |
| NCT of Delhi | North East | 130 | 14 | 116 | 10.8 |
| West Bengal | Bankura | 130 | 14 | 116 | 10.8 |
| NCT of Delhi | South West | 122 | 13 | 109 | 10.7 |
| Karnataka | Tumkur | 131 | 14 | 117 | 10.7 |
| Uttar Pradesh | Hardoi | 113 | 12 | 101 | 10.6 |
| Madhya Pradesh | Ashoknagar | 139 | 14 | 125 | 10.1 |
| Punjab | Tarn Taran | 135 | 13 | 122 | 9.6 |
| Uttar Pradesh | Meerut | 164 | 15 | 149 | 9.1 |
| Tamil Nadu | Madurai | 136 | 12 | 124 | 8.8 |
| Tripura | West Tripura | 138 | 12 | 126 | 8.7 |
| West Bengal | Paschim Medinipur | 98 | 8 | 90 | 8.2 |
| Punjab | Ludhiana | 118 | 9 | 109 | 7.6 |
| Assam | Sonitpur | 133 | 10 | 123 | 7.5 |
| Meghalaya | East Khasi Hills | 131 | 9 | 122 | 6.9 |
| Bihar | Vaishali | 144 | 10 | 134 | 6.9 |
| Andhra Pradesh | Visakhapatnam | 173 | 12 | 161 | 6.9 |
| Meghalaya | West Khasi Hills | 123 | 8 | 115 | 6.5 |
| Bihar | Pashchim Champaran | 137 | 8 | 129 | 5.8 |
| Maharashtra | Aurangabad | 106 | 6 | 100 | 5.7 |
| Tripura | North Tripura | 133 | 7 | 126 | 5.3 |
| Assam | Dibrugarh | 117 | 6 | 111 | 5.1 |
| Karnataka | Dakshina Kannada | 126 | 6 | 120 | 4.8 |
| Assam | Jorhat | 112 | 5 | 107 | 4.5 |
| NCT of Delhi | Central | 115 | 5 | 110 | 4.3 |
| Tripura | South Tripura | 141 | 6 | 135 | 4.3 |
| Assam | Kamrup | 145 | 5 | 140 | 3.4 |
| Odissa | Subarnapur | 150 | 5 | 145 | 3.3 |
| Odissa | Nayagarh | 130 | 4 | 126 | 3.1 |
| Punjab | Patiala | 118 | 3 | 115 | 2.5 |
| Meghalaya | Ribhoi | 125 | 3 | 122 | 2.4 |
| Odissa | Bhadrak | 130 | 3 | 127 | 2.3 |
| Meghalaya | Jaintia Hills | 140 | 3 | 137 | 2.1 |
