# Supplementary material for: Analysis of Cholera Risk in India: Insights from 2017–18 Serosurvey Data Integrated with Epidemiologic data and Societal Determinants from 2015–2019
Source: PLoS Negl Trop Dis. 2024 Sep 3;18(9):e0012450. doi: 10.1371/journal.pntd.0012450 (PMC11398695; doi:10.1371/journal.pntd.0012450)
Supplement: S2 Table — (DOCX) [file pntd.0012450.s002.docx]

**S2 Table: Calculation of Composite Index for all 15 States involved in the study (Description of legends X1, X2... X6 has been given in Figure 1)**

| State | **Elevated vibriocidal titre (≥ 320) (1)** | No. of districts | No. of districts with Cholera | **Population of state** | **Population of cholera affected districts** | **Proportion of population belonged to cholera affected districts (%) (2)** | total cholera outbreak in 5 years | average cholera outbreak in one year  (x) | number of years with cholera outbreak  (y) | **(x)*(y)**  **(3)** | **Proportion of (%) HH with improved sanitation** | **Proportion of (%) HH without improved sanitation**  **(4)** | **Proportion of (%) HH with improved drinking water** | **Proportion of (%) HH without improved drinking water**  **(5)** | **MPI (2015/16)** | **MPI*100**  **(6)** | **Composite Index (1+2+3+4+5+6)** |
| --- | --- | --- | --- | --- | --- | --- | --- | --- | --- | --- | --- | --- | --- | --- | --- | --- | --- |
| Andhra Pradesh | 10.9 | 13 | 1 | 84580777.0 | 4053463.0 | 4.8 | 1 | 0.2 | 1 | 0.2 | 53.6 | 46.4 | 72.7 | 27.3 | 0.065 | 6.5 | 96.1 |
| West Bengal | 12.1 | 19 | 11 | 91276115.0 | 59940811.0 | 65.7 | 32 | 6.4 | 4 | 25.6 | 50.9 | 49.1 | 94.6 | 5.4 | 0.109 | 10.9 | 168.8 |
| Tamil Nadu | 16.7 | 32 | 2 | 72147030.0 | 7723965.0 | 10.7 | 2 | 0.4 | 2 | 0.8 | 52.2 | 47.8 | 90.6 | 9.4 | 0.028 | 2.8 | 88.2 |
| Madhya Pradesh | 13.4 | 50 | 14 | 72626809.0 | 23529164.0 | 32.4 | 30 | 6 | 4 | 24 | 33.7 | 66.3 | 84.7 | 15.3 | 0.18 | 18 | 169.4 |
| Uttar Pradesh | 12.6 | 71 | 1 | 199812341.0 | 2547184.0 | 1.3 | 2 | 0.4 | 2 | 0.8 | 35 | 65 | 96.4 | 3.6 | 0.18 | 18 | 101.3 |
| Rajasthan | 12.6 | 33 | 2 | 68548437.0 | 16674956.0 | 24.3 | 9 | 1.8 | 2 | 3.6 | 45 | 55 | 85.5 | 14.5 | 0.143 | 14.3 | 124.3 |
| Karnataka | 11.5 | 30 | 17 | 61095297.0 | 39492354.0 | 64.6 | 35 | 7 | 5 | 35 | 57.8 | 42.2 | 89.3 | 10.7 | 0.068 | 6.8 | 170.8 |
| Maharashtra | 11.3 | 35 | 16 | 112374333.0 | 46003284.0 | 40.9 | 23 | 4.6 | 5 | 23 | 51.9 | 48.1 | 91.5 | 8.5 | 0.069 | 6.9 | 138.7 |
| Delhi | 9.8 | 9 | 3 | 16787941.0 | 5671970.0 | 33.8 | 7 | 1.4 | 3 | 4.2 | 73.3 | 26.7 | 80 | 20 | 0.016 | 1.6 | 96.1 |
| Bihar | 8.7 | 38 | 0 | 1.0 | 0.0 | 0.0 | 0 | 0 | 0 | 0 | 25.2 | 74.8 | 98.2 | 1.8 | 0.246 | 24.6 | 109.9 |
| Punjab | 8.4 | 20 | 8 | 27743338.0 | 15118506.0 | 54.5 | 30 | 6 | 5 | 30 | 81.5 | 18.5 | 99.1 | 0.9 | 0.025 | 2.5 | 114.8 |
| Tripura | 7.2 | 4 | 0 | 1.0 | 0.0 | 0.0 | 0 | 0 | 0 | 0 | 61.3 | 38.7 | 87.3 | 12.7 | 0.086 | 8.6 | 67.2 |
| Odissa | 5.2 | 30 | 10 | 41974218.0 | 19587830.0 | 46.7 | 12 | 2.4 | 5 | 12 | 29.4 | 70.6 | 88.8 | 11.2 | 0.154 | 15.4 | 161.1 |
| Assam | 5.1 | 27 | 6 | 31205576.0 | 7676324.0 | 24.6 | 11 | 2.2 | 4 | 8.8 | 47.7 | 52.3 | 83.8 | 16.2 | 0.16 | 16 | 123.0 |
| Meghalaya | 4.4 | 7 | 0 | 1.0 | 0.0 | 0.0 | 0 | 0 | 0 | 0 | 60.3 | 39.7 | 67.9 | 32.1 | 0.145 | 14.5 | 90.7 |
