# Supplementary material for: Analysis of Cholera Risk in India: Insights from 2017–18 Serosurvey Data Integrated with Epidemiologic data and Societal Determinants from 2015–2019
Source: PLoS Negl Trop Dis. 2024 Sep 3;18(9):e0012450. doi: 10.1371/journal.pntd.0012450 (PMC11398695; doi:10.1371/journal.pntd.0012450)
Supplement: S3 Table — (DOCX) [file pntd.0012450.s003.docx]

**S3 Table: Districts with reported outbreaks in 3 or more years identified through literature survey**

| **State** | **Districts** | **Number of outbreaks in the year** | | | | | **Total cases** |
| --- | --- | --- | --- | --- | --- | --- | --- |
|  |  | **2015** | **2016** | **2017** | **2018** | **2019** |  |
| Chandigarh | Chandigarh | 5 | 2 | 1 | 2 | 0 | 157 |
| Dadra and Nagar Haveli | Dadra and Nagar Haveli | 2 | 1 | 0 | 1 | 2 | 20 |
| Gujarat | Gandhinagar | 2 | 6 | 2 | 0 | 1 | 571 |
|  | Anand | 1 | 0 | 1 | 1 | 0 | 160 |
|  | Vadodara | 0 | 1 | 1 | 2 | 1 | 199 |
| Maharashtra | Nashik | 0 | 1 | 0 | 1 | 1 | 563 |
| Punjab | Ludhiana | 1 | 0 | 5 | 5 | 0 | 1465 |
|  | Hoshiarpur | 1 | 1 | 0 | 2 | 2 | 590 |
| West Bengal | Purulia | 2 | 7 | 0 | 0 | 1 | 449 |
