# Supplementary material for: Analysis of Cholera Risk in India: Insights from 2017–18 Serosurvey Data Integrated with Epidemiologic data and Societal Determinants from 2015–2019
Source: PLoS Negl Trop Dis. 2024 Sep 3;18(9):e0012450. doi: 10.1371/journal.pntd.0012450 (PMC11398695; doi:10.1371/journal.pntd.0012450)
Supplement: S4 Table — (DOCX) [file pntd.0012450.s004.docx]

**S4 Table: Different ranking scheme against composite score ranking for different states and UTs**

| Column 1 | Column 2 | Column 3 | Column 4 | Column 5 | Column 6 | Column 7 | Column 8 | Column 9 | Column 10 | Column 11 |
| --- | --- | --- | --- | --- | --- | --- | --- | --- | --- | --- |
| State | WASH/ MPI (More score more vulnerability) | vibriocidal titre (≥ 320) | Avg. yearly outbreak*number of outbreak years = **Outbreak Trend** | Composite score | Rank (WASH/ MPI wise) | Outbreak trend wise Rank | vibriocidal titre (≥ 320) rank | Composite score wise rank | Score W/O vibriocidal titre (≥ 320) | Ranking W/O vibriocidal titre (≥ 320) |
| Karnataka | 59.7 | 11.5 | 35 | 170.84 | 3 | 15 | 10 | 15 | 159.3406 | 15 |
| Madhya Pradesh | 99.6 | 13.4 | 24 | 169.39 | 14 | 12 | 14 | 14 | 155.9974 | 13 |
| West Bengal | 65.4 | 12.1 | 25.6 | 168.76 | 7 | 13 | 11 | 13 | 156.6698 | 14 |
| Odissa | 97.2 | 5.2 | 12 | 161.06 | 13 | 10 | 3 | 12 | 155.8663 | 12 |
| Maharashtra | 63.5 | 11.3 | 23 | 138.73 | 6 | 11 | 9 | 11 | 127.4375 | 11 |
| Rajasthan | 83.8 | 12.6 | 3.6 | 124.32 | 9 | 7 | 12 | 10 | 111.7258 | 9 |
| Assam | 84.5 | 5.1 | 8.8 | 122.99 | 10 | 9 | 2 | 9 | 117.8992 | 10 |
| Punjab | 21.9 | 8.4 | 30 | 114.79 | 1 | 14 | 5 | 8 | 106.3942 | 8 |
| Bihar | 101.2 | 8.7 | 0 | 109.9 | 15 | 2 | 6 | 7 | 101.2 | 7 |
| Uttar Pradesh | 86.6 | 12.65 | 0.8 | 101.27 | 12 | 5 | 13 | 6 | 88.62479 | 6 |
| Delhi | 48.3 | 9.8 | 4.2 | 96.08 | 2 | 8 | 7 | 4 | 86.28598 | 4 |
| Andhra Pradesh | 80.2 | 10.9 | 0.2 | 96.09 | 8 | 4 | 8 | 5 | 85.19242 | 3 |
| Meghalaya | 86.3 | 4.4 | 0 | 90.7 | 11 | 2 | 1 | 3 | 86.3 | 5 |
| Tamil Nadu | 60 | 16.7 | 0.82 | 88.20 | 4 | 6 | 15 | 2 | 71.50587 | 2 |
| Tripura | 60 | 7.2 | 0 | 67.2 | 5 | 2 | 4 | 1 | 60 | 1 |

**Note:**

**1. Most vulnerable states - Karnataka, Madhya Pradesh, West Bengal, Odissa, Maharashtra, Rajasthan; least vulnerable states – Tripura, Tamil Nadu, Meghalaya, Andhra Pradesh, Delhi)**

**2.** We ranked the all 15 states without considering the vibriocidal titre (≥ 320) scores (column 11) and compared the rank against the composite score ranks (column 9). It was noted that although the ranks remained almost similar but states with lower seroincidence like Meghalaya, Assam ranked worse in the new scores.
