# Supplementary material for: Analysis of Cholera Risk in India: Insights from 2017–18 Serosurvey Data Integrated with Epidemiologic data and Societal Determinants from 2015–2019
Source: PLoS Negl Trop Dis. 2024 Sep 3;18(9):e0012450. doi: 10.1371/journal.pntd.0012450 (PMC11398695; doi:10.1371/journal.pntd.0012450)
Supplement: S5 Table — (DOCX) [file pntd.0012450.s005.docx]

**S5 Table: Sero-prevalence (%) of vibriocidal antibodies against cholera using optimized cutoff (640) *, in different geographic regions of India, by selected socio-demographic characteristics (n=7882)**

| **Region** | **North** | | **North East** | | **East** | | **West** | | **South** | | **All Region** | |
| --- | --- | --- | --- | --- | --- | --- | --- | --- | --- | --- | --- | --- |
| **Characteristics** | Number Tested | Prevalence (95% CI) | Number Tested | Prevalence (95% CI) | Number Tested | Prevalence (95% CI) | Number Tested | Prevalence (95% CI) | Number Tested | Prevalence (95% CI) | Number Tested | Prevalence (95% CI) |
| **Age Group** | | | | | | | | | | | | |
| 9-17 Years | 819 | 4.8  (3.6- 6.6) | 750 | 7.9  (3.3- 18.0) | 852 | 2.1  (1.1- 3.8) | 759 | 4.8  (2.8- 8.1) | 924 | 4.7  (3.2- 6.8) | 4104 | 4.3  (3.4- 5.5) |
| 18-45 Years | 744 | 5.1  (3.2- 8.2) | 807 | 1.1  (0.3- 3.5) | 729 | 4.6  (2.7- 7.9) | 681 | 5.3  (3.3- 8.2) | 817 | 9.6  (6.0- 14.9) | 3778 | 5.5  (4.3- 7.0) |
| **Sex (9-45 Years)** | | | | | | | | | | | | |
| Male | 721 | 5.4  (2.1- 13.2) | 628 | 3.1  (0.6- 14.4) | 692 | 2.4  (0.9- 6.2) | 664 | 4.7  (2.7- 8.0) | 773 | 5.2  (2.8- 9.3) | 3478 | 4.4  (2.8- 6.6) |
| Female | 842 | 5.1  (3.4- 7.7) | 929 | 2.7  (1.1- 6.5) | 889 | 4.8  (2.9- 7.7) | 776 | 5.4  (3.5- 8.3) | 968 | 10.8  (7.2- 15.8) | 4404 | 5.8  (4.6- 7.2) |
| **Area of residence (9-45 Years)** | | | | | | | | | | | | |
| Rural | 725 | 4.2  (2.7- 6.5) | 786 | 2.9  (0.9- 9.1) | 816 | 3.4  (1.9- 6.2) | 748 | 5.2  (3.4- 7.8) | 898 | 7.7  (4.1- 14.0) | 3973 | 4.6  (3.5- 5.8) |
| Urban | 838 | 6.8  (4.5- 10.2) | 771 | 3.0  (1.5- 5.7) | 765 | 7.6  (5.5- 10.3) | 692 | 4.9  (2.5- 9.5) | 843 | 10.0  (7.5- 13.2) | 3909 | 7.2  (5.7- 9.0) |
| **Overall** | | | | | | | | | | | | |
| 9-45 Years | 1563 | 5.0  (3.6- 7.0) | 1557 | 2.9  (1.0- 8.4) | 1581 | 3.9  (2.4- 6.2) | 1440 | 5.1  (3.6- 7.3) | 1741 | 8.4  (5.6- 12.5) | 7882 | 5.2  (4.2- 6.2) |

*(*Sera samples with >=640 units were considered as positives)*
