# Supplementary material for: Analysis of Cholera Risk in India: Insights from 2017–18 Serosurvey Data Integrated with Epidemiologic data and Societal Determinants from 2015–2019
Source: PLoS Negl Trop Dis. 2024 Sep 3;18(9):e0012450. doi: 10.1371/journal.pntd.0012450 (PMC11398695; doi:10.1371/journal.pntd.0012450)
Supplement: S1 Fig — (The base layer of the map utilized to create this map is taken from open-source platform https://www.indianremotesensing.com/2017/01/Download-India-shapefile-with-kashmir.html). (DOCX) [file pntd.0012450.s006.docx]

**S1 Fig: Different administrative zones of India & the sample locations across each zone**

| **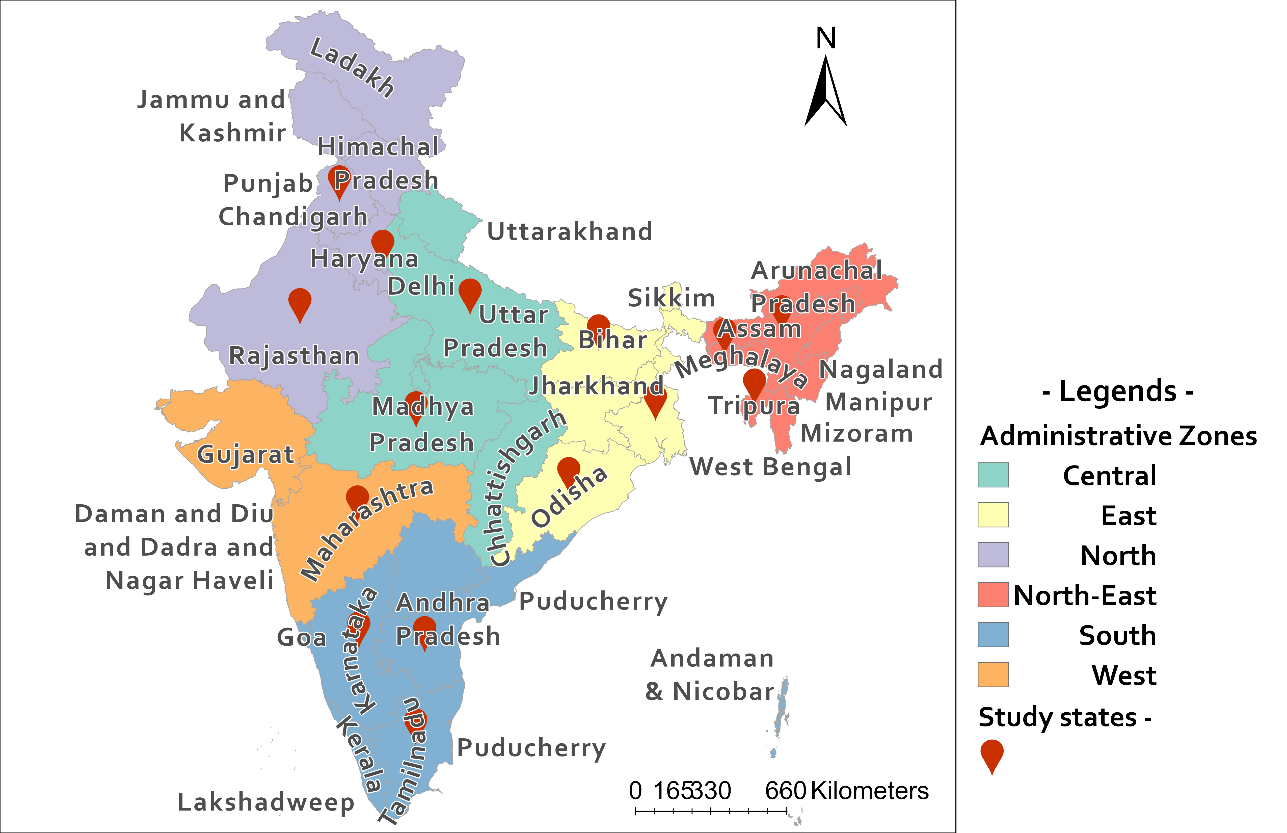** |
| --- |
| **S1 Fig: Different administrative zones of India & the sample locations across each zone**  ***Note:*** *The base layer of the map is taken from open-source platform* [*https://www.indianremotesensing.com/2017/01/Download-India-shapefile-with-kashmir.html*](https://www.indianremotesensing.com/2017/01/Download-India-shapefile-with-kashmir.html) |
