# Supplementary material for: Analysis of Cholera Risk in India: Insights from 2017–18 Serosurvey Data Integrated with Epidemiologic data and Societal Determinants from 2015–2019
Source: PLoS Negl Trop Dis. 2024 Sep 3;18(9):e0012450. doi: 10.1371/journal.pntd.0012450 (PMC11398695; doi:10.1371/journal.pntd.0012450)
Supplement: S2 Fig — (DOCX) [file pntd.0012450.s007.docx]

**S2 Fig:** **Region wise cholera incidence in different districts of India using vibriocidal cut off as 320.**

| 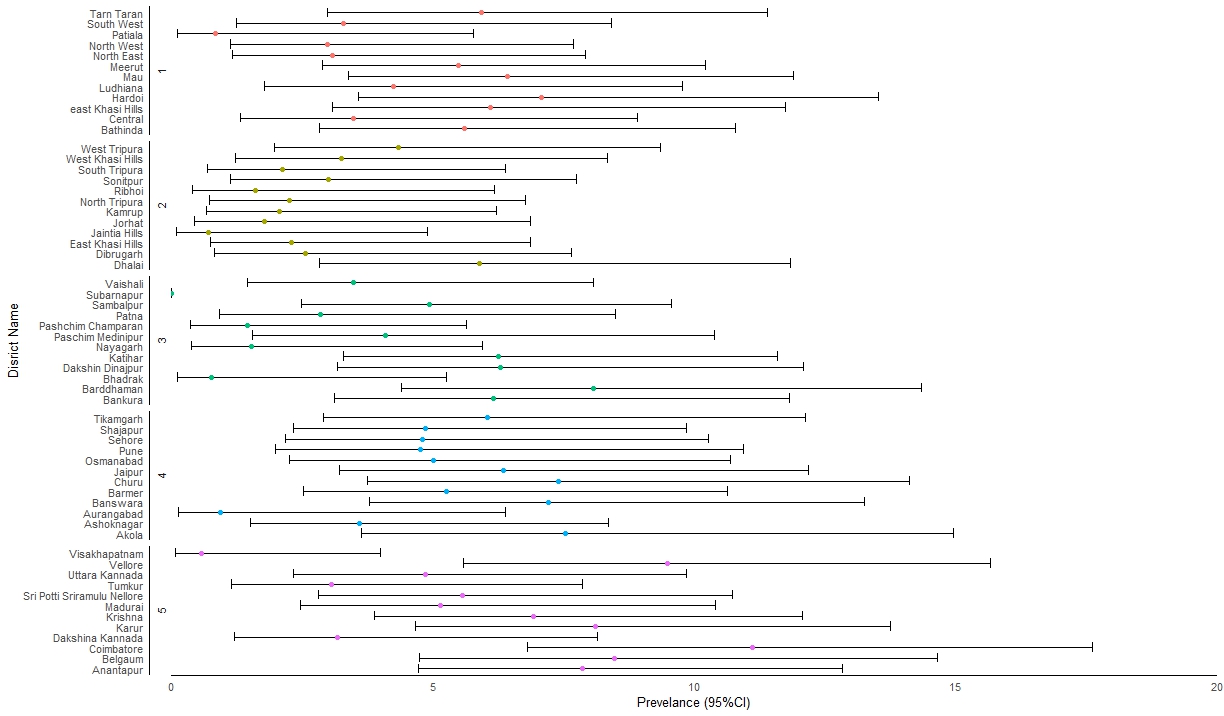 |
| --- |
| **S2 Fig: Region wise cholera incidence in different districts of India using vibriocidal cut off as 320.** |
