# Supplementary material for: Analysis of Cholera Risk in India: Insights from 2017–18 Serosurvey Data Integrated with Epidemiologic data and Societal Determinants from 2015–2019
Source: PLoS Negl Trop Dis. 2024 Sep 3;18(9):e0012450. doi: 10.1371/journal.pntd.0012450 (PMC11398695; doi:10.1371/journal.pntd.0012450)
Supplement: Attachment — Submitted filename: Reviewer report_ Godfrey Bwire.pdf [file pntd.0012450.s008.pdf]

3rd April 2024

Dear Editor PNTD,

Re: Manuscript Number, PNTD-D-23-01663: Understanding the risk of cholera in India through synthesis of serologic, epidemiologic, and societal determinants.

Thank you very much for honoring me and selecting me to review this important manuscript. I am extremely happy to inform you that I have enjoyed this task. Please find my detailed report below. Once again, I greatly appreciate this wonderful opportunity.

Dr. Godfrey Bwire

General comment

This is a well-written study on cholera in India by Kanungo et al. The authors analyzed data on cholera by testing 7,882 stored sera samples collected during 2017-18 from individuals aged 9-45 years. They assessed vibriocidal antibodies to Vibrio cholerae O1 using a cut-off titre of ≥320, defining seroincidence. Additionally, they extracted data from the 2015-19 Integrated Disease Surveillance Programme and published reports.

The authors found that overall, 11.7% (CI: 10.4-13.3%) of the sampled population had seroincident infection. Furthermore, they established that between 2015-2019, the IDSP reported 29,400 cases of cholera across India. These findings are interesting and have the potential to attract actors involved in cholera prevention and research. However, there are several issues that remain unclear in the current version of the manuscript.

Major comments

1. Title, "Understanding the risk of cholera in India through synthesis of serologic, epidemiologic, and societal determinants", This title, as it stands, is open to speculation and does not fully reflect the content of the manuscript. For instance, the data and reports used to generate the manuscript were from the period 2015-2019. I think that the findings of this study would differ from those of a similar study with the same title but using data

from 2020-2023 or reports such as the National Family and Health Survey 2020-2021, https://main.mohfw.gov.in/sites/default/files/NFHS-5_Phase-II_0.pdf. Hence, the authors should revise the title to ensure that it reflect the manuscript content.

2. Lines 89-91, " We generated vibriocidal data using serum samples (n = 7882) collected for the national dengue serosurvey, conducted by ICMR National Institute of Epidemiology (ICMR-NIE), Chennai, India from June 2017 to April 2018.[12]". The period when the sera samples were collected is far back, ie 5-6 years from 2023. Hence, it is possible that the risk factors associated with cholera at that time were addressed. This is reflected in the fact that National Family and Health Survey (NFHS-4, 2015-2016) data was found inadequate for planning by the Indian authorities who subsequently invested in NFHS-5 (2020-2021), https://main.mohfw.gov.in/sites/default/files/NFHS-5_Phase-II_0.pdf. Therefore, the authors need to state this long period past the present time as an important consideration in interpretation of the study findings.

3. Lines 115-116, "at -80°C till accessed for vibriocidal assays. Sera from children aged 5-8 years could not be analysed due to inadequate sample volume". This is an important statement. The omission of children aged 5-8 years due to inadequate sample volume is an issue that may attract attention from actors involved in child protection and rights, such as UNICEF. Therefore, it would be useful if the authors explicitly stated the volume of sera that was deemed adequate. Additionally, they should include in the exclusion criteria a statement indicating that all participants with sera below the stated volume were excluded. Such a statement, in addition to improving the clarity of the information shared, has the potential to encourage researchers to conduct studies that can generate useful and reliable data from small volumes of sera specimens

Other comments

4. Lines 88-93, "The study had been…. " The authors use some abbreviation such as ICMR, for the first time without defining them. The authors need to define all such abbreviations to improve on the manuscript readability.

5. Lines 86-108, the authors frequently use the term 15 states yet India has 28 states and 8 territories, https://en.wikipedia.org/wiki/List_of_Indian_state_days . It would be useful if the information on major administrative units of India was included in this section to enable the readers contextualize the message in the manuscript.

6. Lines 110-111, “In this study, we estimated the seroincidence of cholera among individuals aged 9 to 45 years in the year preceding sample collection in 2017”. This statement could be better if the year was stated e.g in 2016 if that is what the authors meant.
