# Supplementary material for: Analysis of Cholera Risk in India: Insights from 2017–18 Serosurvey Data Integrated with Epidemiologic data and Societal Determinants from 2015–2019
Source: PLoS Negl Trop Dis. 2024 Sep 3;18(9):e0012450. doi: 10.1371/journal.pntd.0012450 (PMC11398695; doi:10.1371/journal.pntd.0012450)
Supplement: Attachment — Submitted filename: Response to Nithya Venkatesan.docx [file pntd.0012450.s009.docx]

**Response to Nithya Venkatesan –**

Thank you for your suggestions. We have addressed and made relevant modifications following the instructions.

| **Editor’s comment** | **Response** | **Changes made on** |
| --- | --- | --- |
| 1) Please ensure that the CRediT author contributions listed for every co-author are completed accurately and in full.  At this stage, the following Authors/Authors require contributions: Suman Kanungo, Ranjan Kumar Nandy, Rounik Talukdar, Manoj Murhekar, Andrew S. Azman, Sonia T Hegde, Pranab Chatterjee, Debjit Chakraborty, Madhuchhanda Das, P Kamaraj, Muthusamy Santhosh Kumar, and Shanta Dutta. Please ensure that the full contributions of each author are acknowledged in the "Add/Edit/Remove Authors" section of our submission form. | **Conceptualization:** SD, SK, & RKN; **Data Curation:** DSG, MM, MSK, RT, DC, PK; **Formal Analysis:** RT, SK, SD, RKN, MM, ASA, PC, DC, MD, MSK, STH; **Funding Acquisition:** SD; **Investigation:** RKN, SK, DC; **Methodology:** SK, RKN, SD, MM, ASA, PC, STH, RT, DC, MD; **Project Administration:** SK, SD, MM, RKN; **Resources:** MM, SK, SD, ASA, MD, MSK; **Software:** SD, RT, SK, PK, MSK; **Supervision:** SK, SD, DSG, ASA; **Validation:** DC, PC, SK, SD, MM, RKN, MD, RT, PK, STH; **Visualization:** RT, PC, PK, MSK; **Writing – Original Draft Preparation:** SK, RT, RKN, DC, ASA, MM; **Writing – Review & Editing:** SK, SD, MM, RKN, PC, DC, MD, PK, MSK, ASA, STH  All the contributing authors have critically reviewed the final version of the manuscript. | Added in the manuscript |
| 2) Please provide an Author Summary | **--** | Added in manuscript |
| 3) Thank you for stating" In this study, we estimated the extent of cholera infection among individuals aged 9 to 45 years corresponding to the year 2016. The samples were collected in 2017 and at a vibriocidal titre of 1: 320, infection occurring in past 365 days could be measured  Please be so kind and clarify whether you have used only the secondary data or used any live participants in this study? | The sera sample collection was done in a different project during national dengue serosurvey, conducted by ICMR National Institute of Epidemiology (ICMR-NIE), Chennai, India from June 2017 to April 2018. Ethical clearance and written verbal consents from the participants were procured regarding the use of the sera samples. The project is published and is reference in our manuscript.  In our study, we utilized the remaining adequate sera samples. Viability of stored sera samples was maintained by archiving samples at -80°C. Frozen aliquots of samples were transported to ICMR-National Institute of Cholera and Enteric Diseases, Kolkata, from ICMR-NIE, Chennai under cold chain, maintaining the required temperature. Received sera aliquots were stored at -80°C till accessed for vibriocidal assays. Sera from children aged 5-8 years could not be analysed due to inadequate (less than 60 µl) sample volume. | The details already exist in the manuscript. |
| 4) Please upload all main figures as separate Figure files in .tif or .eps format. For more information about how to convert and format your figure files please see our guidelines: https://journals.plos.org/plosntds/s/figures   5) Tables should not be uploaded as individual files. Please remove these files and include the Tables in your manuscript file as editable, cell-based objects. For more information about how to format tables, see our guidelines: https://journals.plos.org/plosntds/s/tables   6) We have noticed that you have uploaded Supporting Information files, but you have not included a list of legends. Please add a full list of legends for your Supporting Information files after the references list. | 4) All main figures have been adjusted & modified accordingly, and the same has been uploaded separately in the submission portal  5) Tables are now incorporated within the manuscript as editable, cell-based object.  6) Legend for supporting information file has now been added after the references list. |  |
| 7) Some material included in your submission may be copyrighted. According to PLOSu2019s copyright policy, authors who use figures or other material (e.g., graphics, clipart, maps) from another author or copyright holder must demonstrate or obtain permission to publish this material under the Creative Commons Attribution 4.0 International (CC BY 4.0) License used by PLOS journals. Please closely review the details of PLOSu2019s copyright requirements here: PLOS Licenses and Copyright. If you need to request permissions from a copyright holder, you may use PLOS's Copyright Content Permission form. Please respond directly to this email and provide any known details concerning your material's license terms and permissions required for reuse, even if you have not yet obtained copyright permissions or are unsure of your material's copyright compatibility. Once you have responded and addressed all other outstanding technical requirements, you may resubmit your manuscript within Editorial Manager. Potential Copyright Issues:  i) [Figures 2, and ANNEXURE.docx]. Please (a) provide a direct link to the base layer of the map (i.e., the country or region border shape) and ensure this is also included in the figure legend; and (b) provide a link to the terms of use / license information for the base layer image or shapefile. We cannot publish proprietary or copyrighted maps (e.g. Google Maps, Mapquest) and the terms of use for your map base layer must be compatible with our CC BY 4.0 license. Note: if you created the map in a software program like R or ArcGIS, please locate and indicate the source of the basemap shapefile onto which data has been plotted. If your map was obtained from a copyrighted source please amend the figure so that the base map used is from an openly available source. Alternatively, please provide explicit written permission from the copyright holder granting you the right to publish the material under our CC BY 4.0 license. If you are unsure whether you can use a map or not, please do reach out and we will be able to help you. The following websites are good examples of where you can source open access or public domain maps: * U.S. Geological Survey (USGS) - All maps are in the public domain. (http://www.usgs.gov) * PlaniGlobe - All maps are published under a Creative Commons license so please cite u201cPlaniGlobe, http://www.planiglobe.com, CC BY 2.0u201d in the image credit after the caption. (http://www.planiglobe.com/?lang=enl) * Natural Earth - All maps are public domain. (http://www.naturalearthdata.com/about/terms-of-use/). | 7) i) Figure 2 & annexure 🡪 The base layer of the map for India district boundary shapefile was taken from diva GIS portal. Which is an open-source platform. We are not redistributing the raw shape file data through our manuscript and is adhering to their open-access licence.  The link to the map has been added in figure legend. (https://www.diva-gis.org/gdata) |  |
| 8) Please amend your detailed Financial Disclosure statement. This is published with the article, therefore should be completed in full sentences and contain the exact wording you wish to be published. i). State the initials, alongside each funding source, of each author to receive each grant. | Necessary modifications have been done.  The funding statement now reads as |  |
| 9) In the online submission form you indicate that your data is not available for proprietary reasons and have provided a contact point for accessing this data. Please note that your current contact point is a co-author on this manuscript. According to our Data Policy, the contact point must not be an author on the manuscript and must be an institutional contact, ideally not an individual. Please revise your data statement to a non-author institutional point of contact, such as a data access or ethics committee, and send this to us via return email. Please also include contact information for the third party organization, and please include the full citation of where the data can be found. | We made necessary changes in the manuscript. The contact point for the serosurvey data has been changed to director-niced@icmr.gov.in  The mail does not depict any individual, but is used as a basic contact point for any external queries. |  |
