# Supplementary material for: Analysis of Cholera Risk in India: Insights from 2017–18 Serosurvey Data Integrated with Epidemiologic data and Societal Determinants from 2015–2019
Source: PLoS Negl Trop Dis. 2024 Sep 3;18(9):e0012450. doi: 10.1371/journal.pntd.0012450 (PMC11398695; doi:10.1371/journal.pntd.0012450)
Supplement: Attachment — Submitted filename: Response_to_reviewer_comments_PNTD-D-23-01663R2.docx [file pntd.0012450.s010.docx]

**Response to reviewer comments:**

Respected reviewers, thank you for your suggestions regarding the manuscript. We have tried to address your comments as per the changes mentioned here:

|  | Comments | Responses | Changes made |
| --- | --- | --- | --- |
| Reviewer 4 | | | |
|  | I do not like the model used by the authors since I continue to feel that too much weight it given to the WASH and SES data even though most of the manuscript is taken up by the description of the methods and results of the biocidal antibody testing.  Since vibriocidal results contribute such a small amount to the score, it seems the overall scores might have yielded the same ranking even without including the vibriocidal results.  On line 409 the authors explain "sharing the tool to different experts in the field of cholera and mathematical modelling, it is unanimously agreed as the best possible index with the limited data availability."  I do not think this is an adequate justification.  I believe there should be some additional wording to indicate that this model will need to be evaluated by others to determine its validity (or something to this effect) to indicate this is a limitation. | Respected reviewer, thank you for your suggestions. We have now incorporated the need for validation of the scoring system with more robust dataset in future studies and have added this point to the limitation section. We conducted separate rankings using individual indicators and another without considering vibriocidal scores. These were compared to the overall composite score ranking and detailed in Supplementary Annexure IV. While the composite and non-vibriocidal rankings were largely similar, states with lower disease rates, like Meghalaya and Assam, performed worse in the ranking that excluded vibriocidal data. Further, the WASH/ MPI based ranking was largely dissimilar with composite score ranking (Column 6 Vs. column 9 in Annexure IV) indicating WASH/ MPI data did not unduly affected the composite scores. | Page 15  Line no. 429 – 436  Page 14,  Line no. 411-418 |
| Reviewer 3 | | | |
|  | Results lines 242-245, “(Annexure: Table II). A total of 268 diarrheal outbreaks were reported to the IDSP, five states (West Bengal, Karnataka, Punjab, Madhya Pradesh, and Gujarat) accounted for 60% of these outbreaks. <Figure 2: Year wise reported diarrheal outbreaks from surveyed Indian states & UTs during 2015 - 2019 identified through literature survey>” and  Discussion section Line 263- 266, “Of the 29,400 cases of Acute Diarrheal Diseases (ADDs) reported during confirmed cholera outbreaks, Maharashtra registered the most cases (8,040), followed by West Bengal (5,291). The top five high burden states (Maharashtra, West Bengal, Punjab, Madhya Pradesh, and Gujarat) accounted for 79.6% of all cases, in congruence with prior analyses published in 2017”.  **The authors rightly highlight the significance of acute watery diarrhoea (AWD) as a major public health concern. However, I note a discrepancy in their recommendations, which predominantly focus on cholera while neglecting AWD. To strengthen the manuscript's impact and comprehensiveness, I suggest the authors provide equally robust recommendations for AWD, acknowledging its equal importance in the context of waterborne diseases. By doing so, the authors can ensure a more balanced and effective approach to addressing the burden of diarrheal diseases.** | Respected reviewer, thanks a lot for addressing this important issue. AWD is of utmost importance and is responsible for around 13% mortality among the under 5 children in India (Lakshminarayanan et al. 2015). There is no denial or avoiding of this fact, and numerous research regarding the same is going on nationally or subrationally.  ***Our manuscript was specifically aimed at analysis of Cholera Risk in India utilizing serosurvey, epidemiologic and Societal determinants data. Thereby we did not present extensive recommendations on our own to deal with the cholera disease or acute watery diarrhoea, as this would be out of the scope of this manuscript and would warrant a more comprehensive and separate article/ study.***  While this analysis is focused on cholera, it is possible that similar approaches could be used for other AWD causing pathogens. Looking at cholera specifically allowed us for advocating more concrete, addressable solutions in identified high-risk areas - patterns of AWD may not necessarily be the same as cholera in space and time and thus require a different analysis and possibly recommendations.  Global task force of cholera control charts out definitive steps to fight cholera, and the steps would be applicable for other AWDs too. We have presented the aim of GTFCC in this manuscript and the recommendations which are important and falls within the scope of this manuscript. | -- |
